# PD151 Informal Networking For Health Technology Assessment Capacity Building In African Countries

**DOI:** 10.1017/S0266462324003842

**Published:** 2025-01-07

**Authors:** Jani Mueller, Tara Schuller

## Abstract

**Introduction:**

The HTAfrica group is an informal network of individuals enthusiastic about health technology assessment (HTA) in Africa. In 2021, a grassroots network was founded to explore HTA issues relevant to African countries. Through webinars and information exchanges, the group aims to cultivate social capital and cohesion among members to co-create knowledge on key HTA issues that are important in the region.

**Methods:**

The HTAfrica group uses a social media platform to convene and deliver programming. Individual users request to join and are approved by the group coordinators. Once joined, users can read and make blog posts and participate in webinars offered on relevant topics. Despite the size of the group (177 members in 2024), engagement in 2021 and 2022 was relatively low (average 3.1 people per session). In 2023, a structured eight-part introductory HTA webinar series was offered to improve engagement, in place of the ad hoc discussions on topics that were offered the previous year.

**Results:**

Eight webinars were offered to group members to introduce them to HTA and its potential importance for African countries. They included journal-based case studies of five African countries (Egypt, Ethiopia, Ghana, Malawi, and South Africa) and a concluding webinar to summarize learnings. A co-creation approach was taken where the perspectives of all participants contributed to the group’s learnings. The webinar series ended in June 2024 and had a lower participation rate (average of two per session) but = greater cohesion among participants than did the ad hoc topics approach.

**Conclusions:**

The eight-part series was offered from October 2023 to June 2024. While the number of participants per session was lower in the structured series, the individuals formed stronger connections and gained more intangible benefits. To form a strong network, a mix of both ad hoc hot topics and a structured series approach may be optimal for the formation of looser peripheral parts of the network (ad hoc approach) as well as fostering stronger cohesion in the network core (structured series approach).

